# Exposure-associated DNA methylation among people exposed to multiple industrial pollutants

**DOI:** 10.1186/s13148-024-01705-y

**Published:** 2024-08-20

**Authors:** Chi-Hsin Sally Chen, Tzu-Hsuen Yuan, Tzu-Pin Lu, Hsin-Ying Lee, Yi-Hsuen Chen, Liang-Chuan Lai, Mong-Hsun Tsai, Eric Y. Chuang, Chang-Chuan Chan

**Affiliations:** 1 Institute of Environmental and Occupational Health Sciences, College of Public Health, National Taiwan University, Taipei, Taiwan; 2 Department of Health and Welfare, College of City Management, University of Taipei, Taipei, Taiwan; 3 Institute of Epidemiology and Preventive Medicine, College of Public Health, National Taiwan University, Taipei, Taiwan; 4 Institute of Health Data Analytics and Statistics, College of Public Health, National Taiwan University, Taipei, Taiwan; 5 Graduate Institute of Physiology, College of Medicine, National Taiwan University, Taipei, Taiwan; 6 Institute of Biotechnology, College of Bio-Resources and Agriculture, National Taiwan University, Taipei, Taiwan; 7 Department of Electrical Engineering, College of Electrical Engineering and Computer Science, National Taiwan University, Taipei, Taiwan; 8 Biomedical Technology and Device Research Laboratories, Industrial Technology Research Institute, Hsinchu, Taiwan; 9 Research and Development Center for Medical Devices, National Taiwan University, Taipei, Taiwan; 10 Graduate Institute of Biomedical Electronics and Bioinformatics, National Taiwan University, Taipei, Taiwan

**Keywords:** Petrochemical industry, Heavy metals, PAHs, DNA methylation, SNPs, Gene-environment interaction

## Abstract

**Background:**

Current research on the epigenetic repercussions of exposure to a combination of pollutants is limited. This study aims to discern DNA methylation probes associated with exposure to multiple pollutants, serving as early effect markers, and single-nucleotide polymorphisms (SNPs) as surrogate indicators for population susceptibility. The investigation involved the analysis of urine exposure biomarkers for 11 heavy metals (vanadium, arsenic, mercury, cadmium, chromium, nickel, lead, manganese, copper, strontium, thallium), polycyclic aromatic hydrocarbon (PAHs) (1-hydroxypyrene), genome-wide DNA methylation sequencing, and SNPs array on all study participants. The data were integrated with metabolomics information and analyzed both at a community level based on proximity to home addresses relative to the complex and at an individual level based on exposure biomarker concentrations.

**Results:**

On a community level, 67 exposure-related CpG probes were identified, while 70 CpG probes were associated with urine arsenic concentration, 2 with mercury, and 46 with vanadium on an individual level. These probes were annotated to genes implicated in cancers and chronic kidney disease. Weighted quantile sum regression analysis revealed that vanadium, mercury, and 1-hydroxypyrene contributed the most to cg08238319 hypomethylation. cg08238319 is annotated to the aryl hydrocarbon receptor repressor (AHRR) gene, and AHRR hypomethylation was correlated with an elevated risk of lung cancer. AHRR was further linked to deregulations in phenylalanine metabolism, alanine, aspartate, and glutamate metabolism, along with heightened oxidative stress. Additionally, three SNPs (rs11085020, rs199442, and rs10947050) corresponding to exposure-related CpG probes exhibited significant interaction effects with multiple heavy metals and PAHs exposure, and have been implicated in cancer progression and respiratory diseases.

**Conclusion:**

Our findings underscore the pivotal role of AHRR methylation in gene-environment interactions and highlight SNPs that could potentially serve as indicators of population susceptibility in regions exposed to multiple heavy metals and PAHs.

**Supplementary Information:**

The online version contains supplementary material available at 10.1186/s13148-024-01705-y.

## Introduction

It is estimated that 70–90% of the human disease burden could be attributed to environmental exposures [1]. Traditionally, studies in environmental health have focused on understanding the toxic effects and biological mechanisms related to exposure to single pollutants, such as heavy metals and polycyclic aromatic hydrocarbons (PAHs) [2, 3]. However, real-world scenarios involve humans being exposed to complex mixtures of pollutants simultaneously. Even when individual pollutants do not surpass toxicity or regulatory limits, their cumulative exposure might still lead to additive effects. Once these pollutants enter the body, they interact with different substrates, including genome, epigenome, and metabolome. These interactions are key in determining how an individual responds to pollutants and subsequent health effects. The emerging field of precision environmental health advocates for a comprehensive approach that considers multi-pollutant and integrates multi-omics analysis. This approach aims to achieve a thorough understanding of exposure effects and how they vary from person to person. By examining how multiple pollutants interact with our biological systems, we can gain a deeper insight into their collective impact on overall disease burden [4, 5]. This could be instrumental in identifying individuals who are more susceptible to health issues due to environmental exposures. Such knowledge is essential for creating and implementing evidence-based targeted prevention and intervention strategies.

Residents living near petrochemical complexes could be exposed to multiple industrial pollutants due to the consortium of high pollution facilities including coal-fired power plants and oil refineries. We have conducted a series of studies near the largest petrochemical complex in Taiwan and applied exposomics approach to identify exposure biomarkers, early health effect biomarkers, and metabolomic changes linking multiple-pollutant exposure with multiple adverse health outcomes, including cancer, chronic kidney disease (CKD), liver injuries, hyperlipidemia, and respiratory diseases [6–23]. The exposome is the sum of all the exposures that an individual has from birth to death and exposomics is the comprehensive evaluation of all exposures and their contribution to disease causation or progression. It is recommended in exposomics studies to employ omics methods to identify links between exposures and health outcomes, understand the mechanisms of disease development and progression, and potentially developing new biomarkers for exposure and early health effects [1, 24, 25]. We showed novel omics tools could help identify the complex relationship between well-characterized multiple exposures and health impacts in residents living near a petrochemical complex, but we still lack genomics information. Clarifying the relationship on a genetic and epigenetic level could provide insight on the affected molecular mechanisms and potential public health implications including individual susceptibility to environmental exposures.

Gene-environment interaction is the interplay between gene functions and environmental stress, which could influence phenotypes such as health outcomes. Epigenome has become a focus in gene-environment interactions due to the modifiable characteristics of epigenetic modulators. This suggests a potential role as a biomarker of previous exposures and early effects [4, 5]. DNA methylation is the most extensively studied epigenetic modulator in response to environmental stimuli. Alterations in DNA methylation involves the addition of a methyl group (-CH_2_) to the fifth carbon position of the cytosine base, a process facilitated by the DNA methyltransferases (DNMTs) enzymes [26]. This modification has the potential to influence gene expression and subsequent protein expression without altering the primary DNA sequence. It reflects the organism’s immediate adaptation to environmental exposures, possibly triggered by pollutants exposure stimulating the binding of transcription factors to CpG sites. This, in turn, affects DNMT access and therefore influences gene-specific DNA methylation [27].

Epigenetic studies have investigated heritable alterations in global and gene-specific DNA methylation following exposure to heavy metals such as arsenic (As), cadmium (Cd), nickel (Ni), lead (Pb), mercury (Hg), and chromium (Cr), as well as PAHs. These investigations have revealed associations between these alterations and adverse health effects, including oxidative stress, cardiovascular diseases, cancer, and respiratory diseases [28–33]. However, most of these studies focus on single pollutant exposure, leaving room for exploring exposure to multiple-pollutant mixtures [32]. The identification of gene-specific DNA methylation alterations induced by multiple-pollutant exposure could be used in finding surrogate biomarkers indicative of early health effects.

Individuals carrying distinct single-nucleotide polymorphisms (SNPs) may exhibit varying sensitivity to the toxicity of heavy metal and PAHs exposures [34–36]. SNPs refer to variations in DNA sequences where more than two types of nucleotides can exist at a specific position in DNA, differing among individuals. It is recognized as a useful and widely applicable biomarker to locate genetic distribution and predicting individual responses to specific disease or external stimuli [37–40]. Analyzing SNPs within populations residing in a highly polluted areas could unveil potential genetic markers that could serve as susceptibility biomarkers for risk assessment.

**Fig. 1:**
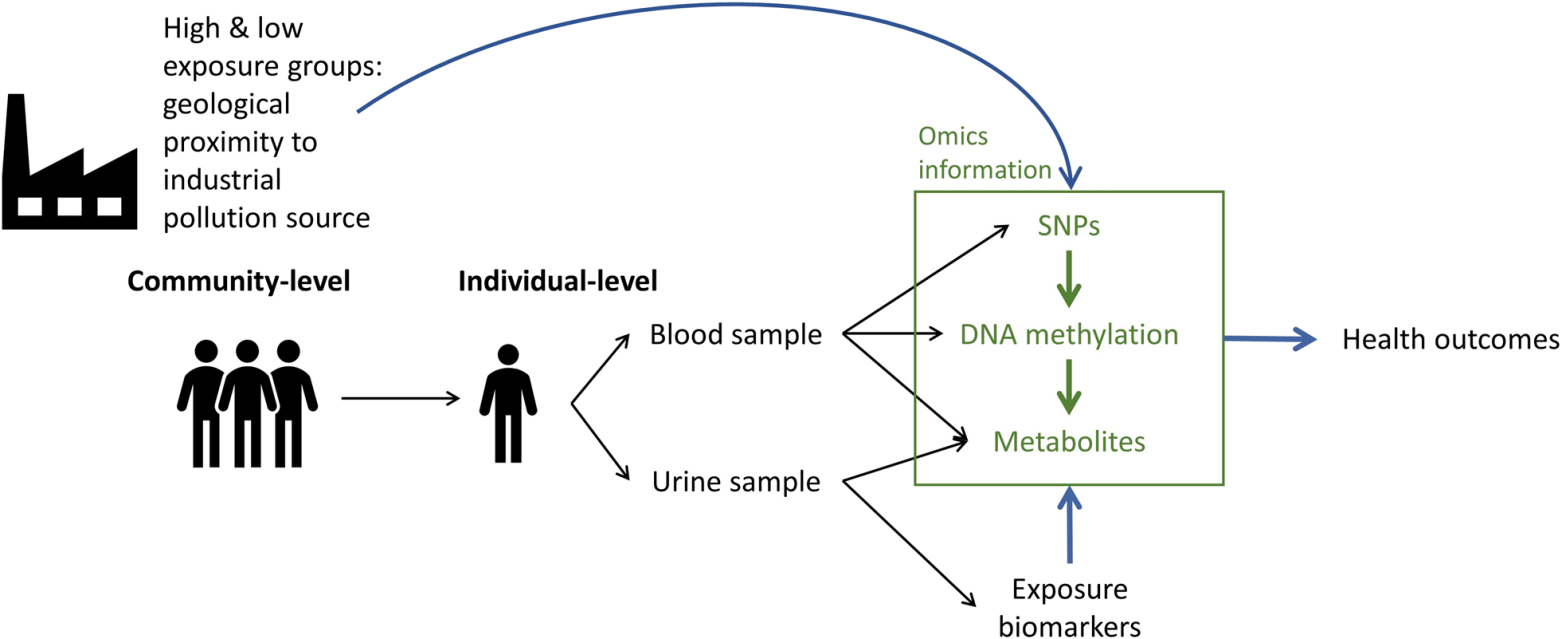
Analytical flowchart of exposure, SNPs, DNA methylation, and metabolite profile on community and individual level

We systematically collected exposure, genetic, epigenetic, and metabolomic information on our study subjects, integrating and analyzing the data on both community and individual levels (Fig. 1). Our objective was to identify multiple-pollutant exposure-related DNA methylation probes, serving as possible markers for early effects, and SNPs that could act as potential surrogate marker for population susceptibility to the health impact of multiple exposures. We then integrated these findings with metabolomics data to strengthen the link between exposure and early health effects observed in residents. We successfully identified exposure-related CpG probes that annotate to genes associated with cancers and chronic kidney disease. Our findings include the aryl hydrocarbon receptor repressor (AHRR), which has been linked to an elevated risk of lung cancer. We further linked AHRR to deregulations in phenylalanine metabolism, alanine, aspartate, and glutamate metabolism, along with elevated oxidative stress. We also identified three SNPs that corresponded to exposure-related CpG probes and showed significant interaction with multiple heavy metals and PAHs exposure. These SNPs have been implicated in cancer progression and respiratory diseases.

Our study employed a precision environmental health approach in a region heavily influenced by a large petrochemical industry. Our SNPs finding could contribute to the identification of individuals at higher risk, and the epigenetic alterations we found may indicate early effects in residents living closer to the petrochemical complex and have been exposed to multiple industrial pollutants. Furthermore, our study enhances the understanding of the impact on critical biological mechanisms that could be precursors to chronic and acute diseases. This comprehensive information offers valuable insights for risk prediction and developing precision prevention and intervention strategies.

## Results

Table 1 shows the basic characteristics, health data, and exposure levels for 159 study subjects. There was no significant difference between high and low exposure groups for age, sex, smoking, drinking, and betel nut chewing history, body mass index (BMI), systolic blood pressure (SBP), alanine transaminase (ALT), aspartate transaminase (AST), high-density lipoprotein cholesterol (HDL-C), and low-density lipoprotein cholesterol (LDL-C). All 12 exposure biomarkers were increased in high exposure group compared to low exposure group. In high exposure group, PAHs exposure biomarker urinary 1-hydroxypyrene (1-OHP) concentrations (0.23 ± 0.56 µmol/mol-creatinine) was significantly elevated compared to low exposure group (0.03 ± 0.01 µmol/mol-creatinine) (*p* < 0.0001). The same trend was found for urinary vanadium (V) (high exposure group: 1.62 ± 1.15 µg/g-creatinine; low exposure group: 0.24 ± 0.11 µg/g-creatinine; *p* < 0.0001). 1-OHP and V had the largest contrast between the two exposure groups with 7.67- and 6.75-fold change in urine concentrations, respectively. Hg had 3.02-fold change (*p* < 0.0001), Cr, manganese (Mn), and Ni with 2.3 (respectively, *p* = 0.011, < 0.0001, 0.024), strontium (Sr) 2.19 (*p* < 0.0001), As 1.90 (*p* < 0.0001), and thallium (Tl) 1.59 (*p* < 0.0001). Pb and copper (Cu) had 1.24- and 1.17-fold change with borderline statistical significance (*p* = 0.054 and 0.057, respectively). Cd was 1.39 times higher in high exposure group compared to low exposure group, but with no statistical significance (*p* = 0.646).

**Table 1:** Comparison of basic characteristics, health data, and exposure levels in 159 study subjects

	High exposure (*n* = 78)		Low exposure (*n* = 81)		*p* value
*Basic characteristics*					
Age, mean ± SD	40.23	± 22.99	40.03	± 22.45	0.955
Sex, *n* (%)	49	(62.82)	46	(56.79)	0.438
Smoke history, *n* (%)	10	(12.82)	13	(16.05)	0.563
Drink history, *n* (%)	10	(12.82)	11	(13.58)	0.888
Betelnut history, *n* (%)	7	(8.97)	11	(13.58)	0.360
BMI, mean ± SD	23.96	± 3.98	23.77	± 4.08	0.766
SBP, mean ± SD	132.70	± 19.84	126.80	± 20.00	0.065
ALT, mean ± SD	26.00	± 24.98	26.62	± 26.00	0.879
AST, mean ± SD	27.62	± 18.53	26.59	± 16.96	0.717
HDL-C, mean ± SD	56.81	± 15.27	52.77	± 14.43	0.088
LDL-C, mean ± SD	105.9	± 32.60	109.1	± 44.20	0.607
*Internal exposures, mean* ± *SD*					
1-OHP	0.23	± 0.56	0.03	± 0.01	< 0.0001
Vanadium (V)	1.62	± 1.15	0.24	± 0.11	< 0.0001
Arsenic (As)	91.43	± 105.45	48.21	± 34.40	< 0.0001
Mercury (Hg)	3.69	± 3.25	1.22	± 0.79	< 0.0001
Cadmium (Cd)	0.82	± 0.92	0.59	± 0.62	0.646
Chromium (Cr)	6.36	± 9.01	2.76	± 1.48	0.011
Nickel (Ni)	9.71	± 14.93	4.24	± 2.50	0.024
Lead (Pb)	0.99	± 0.99	0.80	± 1.04	0.054
Manganese (Mn)	3.23	± 5.17	1.41	± 1.37	< 0.0001
Copper (Cu)	18.33	± 15.01	15.72	± 11.47	0.057
Strontium (Sr)	176.70	± 162.50	80.52	± 51.61	< 0.0001
Thallium (Tl)	0.27	± 0.18	0.17	± 0.13	< 0.0001

Comparison of basic characteristics between the high and low exposure groups for continuous variables was made using Student’s t-test, and for discrete variables, Chi-squared test or Fisher’s exact test. Urinary exposure biomarker concentrations are log-transformed, high and low exposure groups compared by ANCOVA test adjusting age, gender, smoking, alcohol consumption, betel nut chewing, and fish consumption with a post-comparison by Scheffe test. For 1-OHP, unit: µmol/mol-creatinine; for heavy metals, unit: µg/g-creatinine

Despite the significant differences between high and low exposure groups, none of the exposure biomarkers we analyzed exceeded the acute toxicological thresholds. In the high exposure group, the pollutants’ concentration range was approximately equal to or lower than general occupational exposure levels. We had also excluded study subjects who reported to have worked at the petrochemical complex. Therefore, the exposure levels discussed in this study should represent local environmental exposure levels.

**Fig. 2:**
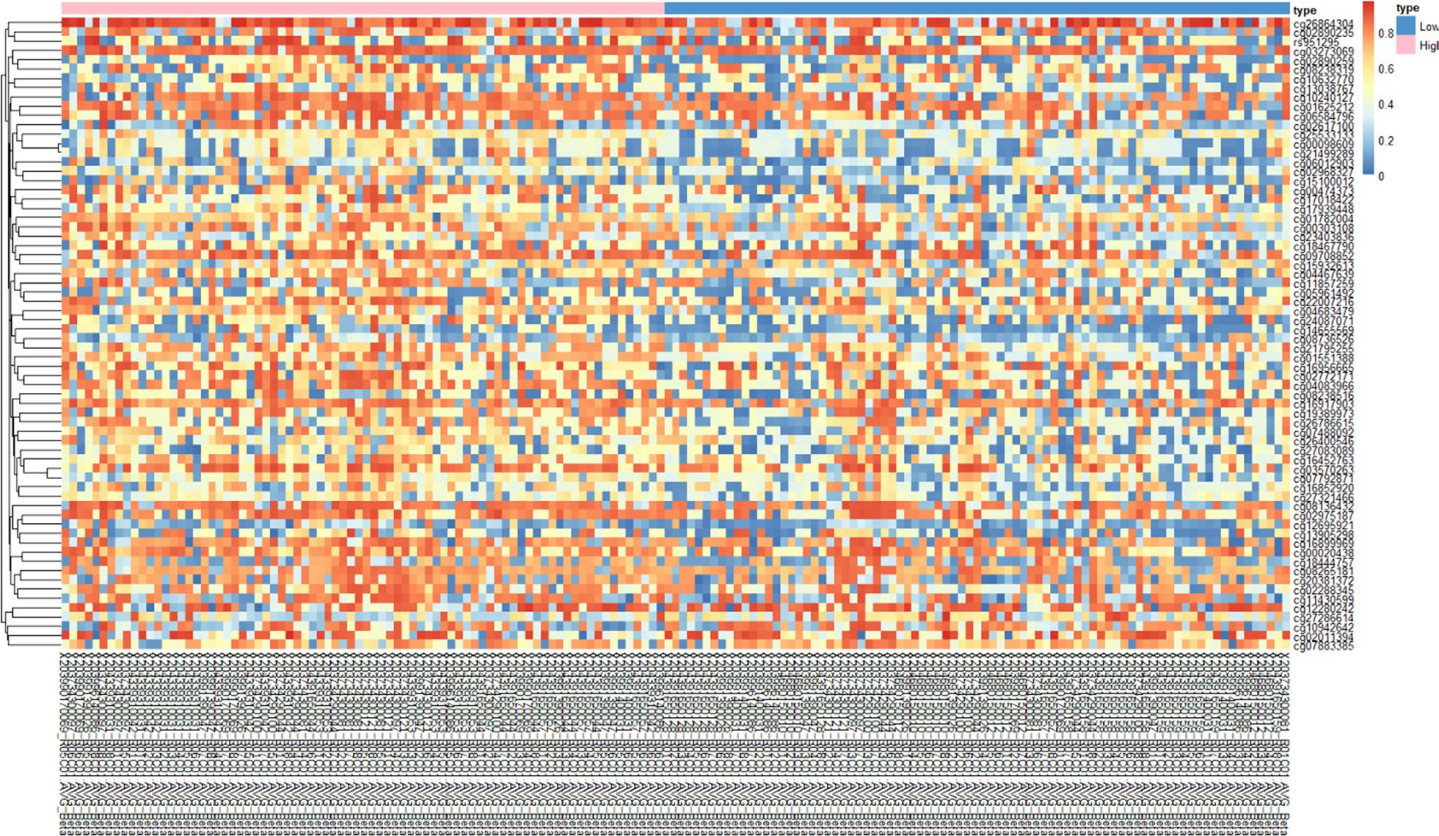
Heatmap of 67 CpGs with significantly different methylation levels between high and low exposure groups

For community-level analysis, we examined the differences between high and low exposure groups for SNPs and DNA methylation. We did not find any significant association between SNPs and exposure status, indicating there are no distinctive genetic differences and no selection bias between our two exposure groups (Figure S5). We did identify 67 probes with DNA methylation levels significantly different between high and low exposure groups (*p* < 0.05 and ∆β >|0.1|), with 62 being hypomethylated and five hypermethylated, corresponding to 41 and 4 known human genes, respectively (Fig. 2; Table 2). These 45 genes were put through the Database for Annotation, Visualization, and Integrated Discovery (DAVID) platform’s functional annotation chart and 16 pathways were found (*p* < 0.05) (Table S2) [41]. These results indicated the multiple-pollutant exposure could impact the epigenomic layer and the probes we identified may potentially serve as surrogate early biomarkers for increased risk of adverse health effects.

**Table 2:** Genes corresponding to CpG probes with DNA methylation levels significantly associated with exposure groups, urine As level, urine Hg level, and urine V level, respectively, in 159 study subjects

	Annotated gene names	
Significant difference in DNA methylation levels between high and low exposure groups		*AHRR, ANTXR1, AOAH, BICD2, C9orf171, CDK18, COPB1, DCHS2, DFNA5, DLG5, FAM193B, FAM47E, FLJ41941, GALNS, HCG9, HLA-B, HSPA12A, HSPB7, HUS1, IL16, KIAA0922, KIAA1199, KIAA1908, LINC00673, LOC100134317, LOC100507443, LOC101927973, MAD1L1, MCF2L, MYBPH, MYO10, NFIC, NSF, PDE11A, PTPRN2, RADIL, RBM41, RNF39, SERPINA10, SLC2A9, STT3A, TBC1D22A, TRAPPC12, TSPY4, YES1*
Significantly associated with urinary As levels		*ALPL, APPL2, ARHGAP8, ATAD2B, BAHD1, C20orf199, C4orf41, CABLES1, CACNA2D2, CAND2, CD81, CDH4, CIT, CPEB4, CPSF3L, CYP2D6, DDX55, DFNA5, DLX2, DYNC2H1, EEF1D, EIF4B, FAM134A, FAM19A5, FBXO31, FKBP3, FOXM1, GNB5, GPATCH2L, GRK5, HDAC2, HIP1, HMX3, KALRN, HMGA1, KIF6, LMTK2, LOC100188947, LYSMD3, Magmas, MANF, MAP4K4, MED13L, MIPEP, NLN, PIK3R3, PRDM6, PRKACB, QKI, RAD51, RCL1, RERE, SERINC4, SLC30A7, SLC35A1, SRD5A3, STAB2, TFG, TRIM31, TSPY4, TTC15, YWHAZ*
Significantly associated with urinary Hg levels		None
Significantly associated with urinary V levels		*AGPAT2, CECR6, CLDN7, CYB5D1, DAXX, DGKG, DNAJB13, DPYSL3, FBXO41, FGF17, FOS, GPR139, GPR35, IGSF9B, KLHL17, LOC84931, LRFN3, MAN2B2, MEX3D, MUM1, MYPN, NAA38, PKMYT1, POLA1, REPIN1, SAMD11, SIPA1L1, SKOR1, SRCIN1, TRPM4, VWA5B1, WDR24*

Underlined: annotated from hypermethylated probes

The 67 CpG probes we identified correspond to 11 SNPs in our SNPs array. We used both quantitative and qualitative models to analyze whether these 11 SNPs are indeed associated with the methylation alteration of the corresponding probes. We then applied a linear regression model to investigate whether the interaction effect between exposure status and SNPs has significant influence on DNA methylation levels. We found in quantitative model all 11 SNPs were significantly associated with methylation alteration of corresponding CpG probes (*p* < 0.05). Linear regression analysis showed SNPs rs11085020 in Nuclear Factor I C (NFIC) and rs199442 in N-ethylmaleimide-sensitive factor (NSF) had significant interaction effects with exposure status that influenced DNA methylation level of corresponding CpG probe (*p* < 0.05) (Figures S6A and B). In qualitative model, all 11 SNPs were significantly associated with the methylation alterations of corresponding CpG probes (*p* < 0.05), and when the SNP variable was coded based on whether the minor allele was carried, rs10947050 in Ring Finger Protein 39 (RNF39) had significant interaction effects with exposure status that influenced DNA methylation level of corresponding CpG probe (*p* < 0.05) (Fig. S6C). However, when SNP variable was coded based on whether the major allele was carried, linear regression analysis did not identify any significant interaction effects between SNPs and exposure status. These results identified SNPs that could potentially serve as a surrogate marker for susceptibility.

We found that out of the 67 exposure-related CpG probes, 40 had DNA methylation levels significantly associated with at least one exposure-related metabolite we previously identified (*p* < 0.01) [11–13]. Methylation level of CpG probe cg01625212 was significantly associated with 9 exposure-related metabolites (7 urinary metabolite and 2 lipids), cg10632770 with 6 urinary metabolites, cg08238319 with 3 urinary and 2 serum metabolites (5 in total), cg21499289 with 5 urinary metabolites, cg00303108 with 4 metabolites (1 urinary, 1 lipid, and 2 serum metabolites), and cg02617100 with 4 urinary metabolites (Table 3). For cg01625212, 3 out of the 9 associated metabolites, palmitic acid, myristic acid, and stearic acid, are involved in fatty acid biosynthesis pathway. Cg21499289 (corresponding gene C9orf171) and cg02617100 (corresponding gene LINC00673) were also associated with both palmitic acid and stearic acid. Cg08238319 (corresponding gene AHRR) was associated with phenylalanine, an important exposure-related intermediate biomarker, as well as γ-Aminobutyric acid and oxoglutaric acid, both involved in alanine, aspartate, and glutamate metabolism, an exposure-related pathway we identified in our previous study. These results suggest a link from DNA methylation alterations caused by multiple exposure affected to biological mechanisms and early health effects we previously identified through metabolomics studies.

**Table 3:** Association between exposure-related CpG probes and exposure-related metabolites

CpG probe^1^	cg01625212	cg10632770	cg08238319	cg21499289	cg00303108	cg02617100
Annotated gene	None	KIAA1199	AHRR	C9orf171	RBM41	LINC00673
*Urinary metabolite*^2^						
(S)-3-Hydroxyisobutyric acid	0.002					
2-Ethylhydracrylic acid			0.004			
2,4-Dihydroxybutanoic acid				0.008		0.005
Acetoin	< 0.001					
Diacetone alcohol		0.004				
Dodecane		0.004				
Glyceric acid		0.006				
Hypoxanthine		0.001				
Inositol						0.010
Myristic acid	0.003					
Palmitic acid	0.003			0.002		0.009
Phenol	0.002					
Phenylalanine			0.003			
Rhamnose	< 0.001					
Serine					0.007	
Stearic acid	< 0.001			0.003		0.005
Thiodiacetic acid		0.009				
Tridecane		0.009		0.007		
Uracil				0.003		
γ-Aminobutyric acid			0.003			
*Serum metabolite*^3^						
Carnitine			0.006			
Inosine					0.007	
Oxoglutaric acid			0.002			
Pyroglutamic acid					0.002	
*Serum lipid*^4^						
LPC (18:1/0:0)	0.003					
PC (18:2/20:5)					0.002	
SM (d18:1/25:0)	< 0.001					

*p* values are shown

^1^Only CpG probes with at least four significantly associated metabolites are included

^2^*N* = 49 (high exposure group *N* = 21; low exposure group *N* = 28)

^3^*N* = 43 (high exposure group *N* = 23; low exposure group *N* = 20)

^4^*N* = 44 (high exposure group *N* = 20; low exposure group *N* = 24)

For individual-level analysis, Pearson’s correlation analysis identified 70 probes with DNA methylation levels associated with As urine concentration, 2 with Hg, and 46 with V (*p* < 1 × 10^−5^), corresponding to 62, 0, and 32 known human genes, respectively (Table 2). Interestingly, for As, the associated probes were mostly hypomethylated, while for V it was the opposite. This suggests different pollutants could have varying effects on DNA methylation. When we compare these results with community-level findings, only two genes significantly different between high and low exposure groups were also associated with urinary As concentrations (DFNA5、TSPY4). Gene-Set Enrichment Analysis (GSEA) pathway analysis results showed four pathways related to urinary As and Hg exposure levels each (*p* < 0.05), while no pathways were identified to be related to V exposure levels (Table S3) [42].

**Fig. 3:**
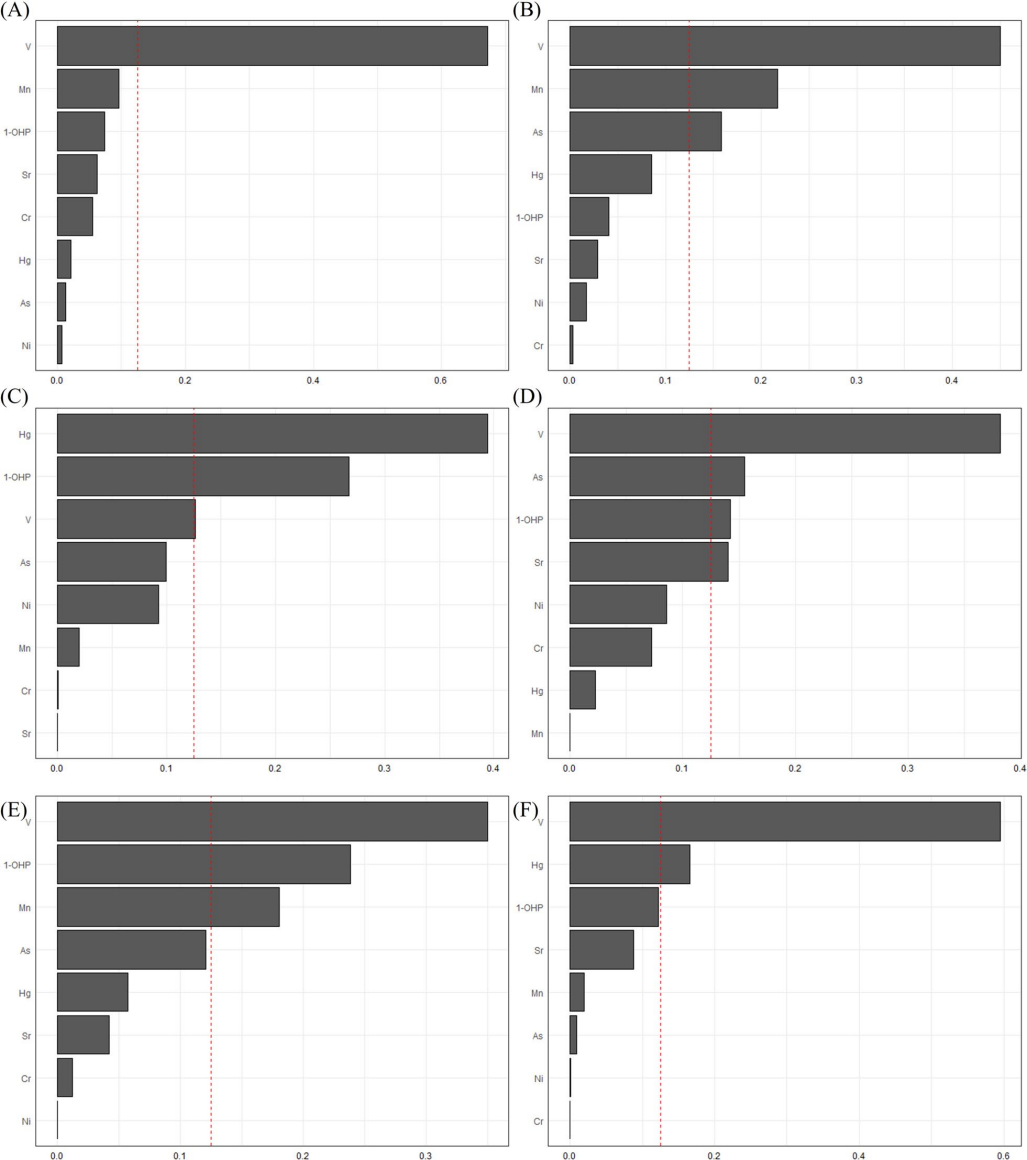
Combined associations between biomarkers V, 1-OHP, Hg, Cr, Mn, Ni, Sr, and As with CpG **A** cg01625212 (*p* = 0.006), **B** cg10632770 (*p* = 0.011), **C** cg08238319 (*p* < 0.001), **D** cg21499289 (*p* = 0.010), **E** cg00303108 (*p* = 0.009), and **F** cg02617100 (*p* < 0.001) based on weighted quantile sum (WQS) regression analysis in 159 study subjects

We employed weighted-quantile sum (WQS) regression for multi-pollutant analysis at an individual level. Figure 3 shows the association between the mixture of eight exposure biomarkers with the highest fold-change between high and low exposure groups (V, 1-OHP, Hg, Cr, Mn, Ni, Sr, and As) and the six exposure-related CpG probes that were linked to exposure-related metabolites (cg01625212, cg10632770, cg08238319, cg21499289, cg00303108, and cg02617100), respectively. For all six CpG probes, the association was statistically significant (*p* < 0.05), and V was the main contributor except for cg08238319. In Fig. 3A, V predominated the mixture index for cg01625212 as the largest (weight = 0.65) contributor to the mixture effect (cutoff weight defined as 0.125, the inverse of the number of variables included in the mixture) (*p* = 0.006). For cg10632770, V (weight = 0.45), Mn (weight = 0.22), and As (weight = 0.16) were the major contributors to the index (*p* = 0.011) (Fig. 3B). Hg was the main contributor for cg08238319 (weight = 0.39) with 1-OHP (weight = 0.27) (*p* < 0.001) (Fig. 3C). In cg21499289, V (weight = 0.38), As (weight = 0.15), 1-OHP (weight = 0.14), Sr (weight = 0.14) reached the cutoff weight for significant contribution (*p* = 0.010). V (weight = 0.35), 1-OHP (weight = 0.24), and Mn (weight = 0.18) contributions were most significant in the mixture effect for cg00303108 (*p* = 0.009) (Fig. 3E). Figure 3F shows that for cg02617100, V contributed to over half of the mixture index (weight = 0.59) followed by Hg (weight = 0.17) (*p* < 0.001).

Our results identified three SNPs that could potentially act as surrogate markers for susceptibility to multiple-pollutant exposure. We also found DNA methylation probes that could be affected by multiple-pollutant exposure, suggesting they could serve as possible surrogate biomarkers for early health effects. Additionally, we discovered DNA methylation alterations that could link multiple-pollutant exposure to metabolic changes and early health effects, including oxidative stress. However, due to the limited sample size, further studies are required to validate these findings.

## Discussion

This is the first study to apply a multi-omics approach to investigate DNA methylation and SNPs profiles in industrial area residents chronically exposed to multiple pollutants. Previous studies mainly focused on single toxic exposure or specific SNPs and CpG sites. Through a comprehensive analysis of various omics layers, our study epitomizes the principles of precision environmental health [4]. We have identified and linked potential underlying individual risk factors (SNPs) and exposures (multiple pollutants) that contribute to early health effects with specific molecular endotypes (DNA methylation alterations and exposure-related metabolites). Our findings contribute to the understanding of the health impacts on the local community and population and could guide future prevention and intervention strategies.

Of the 45 differentially methylated genes we identified in community-level analysis, only aryl hydrocarbon receptor repressor (AHRR) had been reported in epigenetic studies for toxic exposure. Smoking-related Cd exposure and occupational PAHs exposure have been significantly associated with differentially methylated CpGs that annotate to AHRR gene [43–45]. In Taiwan, Tantoh et al. reported non-smoking adults living in areas with higher PM_2.5_ level had lower AHRR methylation [46].

Previous studies reported correlation between AHRR methylation and lung cancer risk, and Jacobsen et al. suggested adding AHRR (cg05575921) methylation as an eligibility criterion could enhance the specificity of low-dose computer tomography (LDCT) lung cancer screening [47]. We did not identify the same CpG probe cg05575921 in our study; however, we did find cg08238319 which also annotated to AHRR and was significantly hypomethylated in high exposure group compared to low exposure group (Table 2). There was no significant difference in smoking history between our high and low exposure groups (Table 1). However, due to the small sample size of our study subjects, we could not rule out the possible effects of smoking on AHRR methylation in our study subjects. We did establish connection between combined exposure biomarkers and cg08238319 DNA methylation in our study subjects, with Hg contributing to almost half the mixture index, followed by 1-OHP and V (Fig. 3C). Previous studies had not reported Hg and V exposure to be associated with AHRR methylation. Interestingly, we also did not identify association between Hg and AHRR or V and AHRR through individual-level analysis, which further suggest that it was the combined effect of multi-pollutant exposure that influenced AHRR methylation (Table 2).

We further linked AHRR methylation to five previously identified exposure-related metabolite features: 2-ethylhydracrylic acid, phenylalanine, γ-Aminobutyric acid, carnitine, and oxoglutaric acid (Table 3). We had reported in the same study area that As, Cd, and Ni exposure could affect phenylalanine metabolism pathway, and phenylalanine could be linked to oxidative stress biomarkers 8-OHdG, HNE-MA, 8-isoPGF_2α_, and 8-NO_2_Gua [11]. In the same study, we showed PAHs, As, Cu, Cd, Ni, and Hg exposure was associated with γ-Aminobutyric acid and oxoglutaric acid from alanine, aspartate, and glutamate metabolism and could also be linked to increased oxidative stress. Our findings suggest a potential gene-environment interaction from multi-pollutant exposure to AHRR DNA hypomethylation that could be further linked to deregulations in phenylalanine metabolism, alanine, aspartate, and glutamate metabolism, and elevated oxidative stress.

For the other differentially methylated genes we identified in community-level analysis, several have been reported in cancer studies including DFNA5, KIAA1199, and LINC00673 which were all hypomethylated in our high exposure group compared to low exposure group. DFNA5 methylation has been suggested as a biomarker for breast cancer, colorectal cancer, and gastric cancer [48–50]. Kuscu et al. reported KIAA1199 expression is upregulated in breast cancer through DNA methylation regulatory mechanisms [51]. LINC00673 is highly expressed in prostate cancer tissues [52]. MAD1L1 was hypermethylated in our study, and in previous studies methylation of MAD1L1 was negatively associated with cancer incidence [53]. For other diseases, DNA methylation differences for PTPRN2 was found in CKD patients [54]. We had previously reported elder and female residents living in the high exposure area had increased carcinogenic exposure and elevated risk of all cancers after the complex had been operating for 10 years [15, 16]. We also found increased risk of CKD associated with proximity to the complex and urine As and 1-OHP levels [18, 19]. These findings suggest the DNA methylation probes we identified could be considered as potential biomarkers for cancer and CKD after multiple-pollutant exposure.

For individual-level analysis, we found DNA methylation of 62 known human genes, including FOXM1, HDAC2, and SATB2, were associated with As urinary concentrations (Table 2). This supports previous studies that showed As exposure led to upregulation of gene transcription for proto-oncogene FOXM1, deregulation of HDAC2 protein levels, and overexpression of SATB2 in cell models [55–57]. Hg urine concentrations did not correspond to any known human genes, and although urinary levels of V were associated with DNA methylation of 32 genes, we did not find any previous studies reporting similar findings. This suggests that the DNA methylation probes we found could provide insight in understanding the epigenetic changes induced by heavy metals exposure.

There was little overlap between the exposure-related CpG probes identified through community-level analysis and individual-level analysis (Table 2). However, the CpG probes we identified through community-level analysis were significantly associated with the mixture of exposure biomarkers with major contributions from V, 1-OHP, Hg, and As (Fig. 3). This corresponded with our previous study where Hg and V were most prominent in the mixture effect associated with oxidative stress biomarkers [12, 13]. Our findings showed the difference between using multi-pollutant model and single pollutant models to identify epigenetic changes, reaffirming the importance of acknowledging and considering the combined effect of simultaneous exposure to multiple pollutants in real-world settings.

The three SNPs we identified that corresponded to exposure-related CpG sites with significant interaction effects with exposure status: rs11085020 in NFIC, rs199442 in NSF, and rs10947050 in RNF39 have not been reported in previous studies. However, NFIC is a transcription factor that plays a role in cell proliferation, differentiation, and migration during organ development and has been reported as a tumor suppressor gene in breast carcinomas, osteosarcoma, and T-cell lymphomas [58]. Lee et al. suggest through animal model results that NSF mediates inflammation responses in respiratory disease [59]. These SNPs we found could potentially serve as genetic markers for health-related risk evaluation in populations living in this area, especially based on our previous reports all cancers in elder female residents and respiratory diseases in children and adolescents [15, 22].

Although the exposure levels detected in this study does not exceed acute toxicological thresholds, heavy metals could still accumulate and/or have synergistic effects in the body after exposure. Residents living near petrochemical industrial areas are likely to experience long-term and stable low-dose exposure to heavy metals due to the continuous operation of these complexes. Previous studies we conducted in this area have shown that even at such low exposure levels, significant correlation between the level of exposure and health effects can still be observed, including early health effects such as increased oxidative stress and alterations in metabolite profiles in high exposure group compared to low exposure group [11–13].

There are limitations to this study. Firstly, we applied genome-wide DNA methylation sequencing analysis which still has the possibility of inaccurate identification and we could not provide exact quantification of DNA methylation levels. Secondly, to maximize the possibility to identify potential loci, we used a loose *p* value threshold. However, it allowed us to extend the coverage of more loci. Lastly, our study subjects were selected from a prospective cohort of 3230 participants. Due to sample availability and required sample quality for multi-omics analyses, our sample size was limited. However, our selection criteria minimized bias and ensured representation of the cohort. Additionally, it is a cross-sectional study and therefore we were unable to verify the stability of the quantitative changes we identified and thus large samples size is required to further validate the findings before its applications. It is also possible that other confounding factors such as preexisting physical conditions, dietary habits, and occupational exposures could have influenced exposure levels, DNA methylation, and metabolomics results. We minimized these biases by selecting participants with no prior chronic diseases and no prior work experience at the pollutants’ main emission source, No. 6 Naphtha Cracking Complex, according to their interview-administered questionnaire surveys.

## Conclusion

In this study, we applied a precision environmental health approach in a highly polluted industrial community and identified DNA methylation probes that could serve as surrogate markers for early effects of multiple-industrial-pollutant exposure, three SNPs that could potentially be used to identify vulnerable populations more susceptible to multiple-industrial-pollutant exposure, and gene-environment interactions that linked multiple-pollutant exposure with epigenetic changes and biological pathways related to adverse health outcomes such as increased oxidative stress and cancer. Our findings characterized the complexity of exposure and health impacts and can provide information for risk prediction models and the development of precision prevention and intervention strategies in this area.

## Materials and methods

### Study area and subjects

Our study comprised 159 subjects selected from a prospective cohort of 3,230 participants who had resided in communities surrounding No. 6 Naphtha Cracking Complex for at least five years. All subjects completed interview-administered questionnaire surveys to gather information on age, gender, smoking, alcohol consumption, and betelnut chewing habits. Additionally, they underwent a health examination, including measurements of height, weight, and blood pressure.

Each participant provided a morning spot urine sample for the analysis of exposure biomarkers and a fasting blood sample to measure ALT, AST, HDL-C, and LDL-C.

We categorized the study area based on the proximity of home addresses to the complex, resulting in a high exposure community (three townships closest to the complex) and a low exposure community (seven townships further away) (see Fig. S1).

We used urine exposure biomarkers, specifically V levels and PAHs exposure biomarker 1-OHP, along with home addresses to define the high and low exposure groups. Out of our 159 study subjects, 78 residing in the high exposure community, with urinary 1-OHP and V levels in the top 60% of the cohort, were identified as the high exposure group. The remaining 81 subjects, living in the low exposure community with urine concentrations of 1-OHP and V in the bottom 40% of the cohort, were identified as the low exposure group.

The No. 6 Naphtha Cracking Complex commenced operations in 1999 and is situated in Yunlin County on the west coast of central Taiwan, covering a total area of 2,603 hectares. The complex comprises 53 plants, including a coal-fired power plant with a total capacity of 1.8 million kW, oil refinery plants processing 530,000 barrels of crude oil per day, three naphtha cracking plants producing 2.935 million tons of ethylene annually, and co-generation plants with a capacity of 2.75 million kW [60].

The prospective cohort used in this study was recruited from 2009 to 2011. Approval for this study was granted by the Research Ethics Committee of the National Health Research Institutes (accession number: 201704053RIND), and informed consent was obtained from each participant.

### Internal exposure

Urinary levels of V, As, Hg, Cd, Cr, Ni, Pb, Mn, Cu, Sr, and Tl were analyzed using inductively coupled plasma mass spectrometry (ICP-MS) and 1-OHP using high-performance liquid chromatography (HPLC) following previously reported methods [9–11]. Standard reference materials were used to confirm accuracy (SERO, Billingstad, Norway). In each experiment batch, we ensured the relative error of ten spiked samples was below 10% for measurement stability. Batches with a recovery rate lower than 85% were reanalyzed.

The method detection limit (MDL) for each exposure biomarker was 0.016 (V), 3.325 (As), 0.440 (Hg), 0.129 (Cd), 0.155 (Cr), 1.204 (Ni), 0.300 (Pb), 0.060 (Mn), 1.444 (Cu), 3.920 (Sr), and 0.041 (Tl) μg/L. 1-OHP analysis had an MDL of 0.01 ng/mL with an 89.6% recovery rate, and the coefficient of variation was 4.0% for repeated measurements. Urine concentration of exposure biomarkers below the MDL was replaced by half of the MDL for data analysis.

To minimize batch variations, we included the analysis of standard tune solution in each batch before sample detection to ensure instrument stability and adjust signal quantification. National Taiwan University Hospital medical diagnosis laboratory analyzed urinary creatinine by enzyme-linked immunosorbent assay, and we used the creatinine concentrations to adjust urinary exposure biomarker levels. All urine samples underwent creatinine analysis, and samples with urinary creatinine concentrations below 30 or above 300 mg/dL were excluded from further data analysis due to potential abnormalities of unknown reasons.

### DNA extraction

Two hundred microliters of whole blood was utilized for DNA extraction in each study subject, employing the QIAamp Blood Mini Kit (QIAGEN). The extracted DNA concentration must exceed 100 ng/μL, with an A260/A280 ratio between 1.6 and 2.0, A230/A260 greater than 1.6, A320 near 0, and levels of fragmentation checked via electrophoresis. These criteria ensure compliance with the quality standards required for DNA methylation and SNP analysis.

### SNPs analysis

The DNA from each study subject was individually adjusted to a concentration of 15 ng/μL, and 50 μL of each individual's sample was then placed into ABgene 96-well plates. These prepared samples were sent to the National Center for Genome Medicine for analysis using the Affymetrix Axiom genome-wide array TWB 2.0 (Thermo Fisher Scientific), which contains 752,921 probes.

### DNA methylation analysis

Five hundred nanograms of DNA samples from each study subject was used for genome-wide DNA methylation sequencing analysis. Samples were prepared using the Illumina Infinium Human MethylationEPIC BeadChip platform following the manufacturer's standard protocol. Subsequently, the prepared samples were scanned with Illumina HiScan (Illumina, Inc.), and DNA methylation levels were analyzed using GenomeStudio software v2011.1. The analysis covered more than 850,000 CpG sites.

### Exposure-related metabolite features

Exposure-related urinary metabolite features, serum metabolite features, and serum lipid features were identified in previous studies [11–13]. A total of 103 metabolite features were included for data integration and analysis, comprising 76 urinary metabolite features, 9 serum metabolite features, and 18 serum lipid features (Table S1). Of the urinary metabolites, data were available for 49 study subjects, including 21 from the high exposure group and 28 from the low exposure group. Serum metabolite data were available for 43 study subjects (high exposure group *N* = 23, low exposure group *N* = 20), and lipid information was available for 44 study subjects, with 20 in the high exposure group and 24 in the low exposure group.

### Community-level analysis

We first examined the differences between high and low exposure groups across various parameters, including basic characteristics, urinary exposure biomarkers, SNPs, and DNA methylation. To compare basic characteristics, we used Student’s t-test for continuous variables and Chi-squared test or Fisher’s exact test for discrete variables. For exposure biomarkers, urinary concentrations underwent log transformation and were subsequently compared using ANCOVA, adjusting for age, gender, smoking, alcohol consumption, betel nut chewing, and fish consumption. Post-comparisons were made using Scheffe test. For SNPs array data, a Pearson correlation test was applied to clarify the association between SNPs and exposure. For DNA methylation, all β-values from the microarrays were first transformed into M-values for improved consistency and robustness. Wilcoxon’s rank sum test was performed to identify exposure-related probes, considering the probes with *p* < 0.05 and ∆β >|0.1| as significant (Figure S2). To further explore the biological relevance, gene annotation was performed to identify known human genes corresponding to the exposure-related probes. The identified genes were then put through pathway analysis using the Database for Annotation, Visualization, and Integrated Discovery (DAVID) platform [41].

Association between SNPs and DNA methylation level of corresponding CpG probes was examined using Fisher’s exact test and Wilcoxon’s rank sum test. Additionally, a linear regression model was applied to investigate whether the interaction effect between SNPs and exposure status has significant influence on DNA methylation levels (Fig. S3). These analyses were conducted under both quantitative and qualitative models. In quantitative model, an SNP variable would be coded as “0”, “1”, or “2” based on the allele pair, while in the qualitative model, the SNP variable were encoded as “0” or “1” depending on whether the corresponding allele was carried (Figure S4). For Fisher’s exact test, study subjects were categorized into two DNA methylation groups using two distinct approaches: hypermethylated (β < 0.3)/unchanged (β ≥ 0.3) or hypomethylated (β > 0.7)/unchanged (β ≤ 0.7) (Figure S4).

To ascertain the association between exposure-related CpG probes and exposure-related metabolites, we conducted Pearson’s correlation analysis to examine the significance of difference between high and low exposure groups (*p* < 0.01).

### Individual-level analysis

Pearson’s correlation analysis was applied to identify DNA methylation probes exhibiting a significant association to urinary As, Hg, and V concentrations, respectively (*p* < 1 × 10^−5^). All 865,918 CpG probes from the DNA methylation microarray along with individual urine As, Hg, and V concentrations data were put through Gene-Set Enrichment Analysis (GSEA) for pathway analysis by random walk approach [42].

For multi-pollutant analysis at an individual level, WQS regression analysis was applied to analyze the association between combined exposure biomarkers with CpG probes, using 100 bootstrap samples (*p* < 0.05).

### Supplementary Information

Additional file1.

## Data Availability

The datasets used and/or analyzed during the current study are available from the corresponding author on reasonable request.
