# Supplementary material for: Exposure-associated DNA methylation among people exposed to multiple industrial pollutants
Source: Clin Epigenetics. 2024 Aug 20;16:111. doi: 10.1186/s13148-024-01705-y (PMC11337639; doi:10.1186/s13148-024-01705-y)
Supplement: Supplementary file 1 — Additional file1. [file 13148_2024_1705_MOESM1_ESM.docx]

**Supplementary Information**

Figure S1. GIS map of (A) Yunlin County in central Taiwan and (B) location of high and low exposure areas and petrochemical plants.

Figure S2. Analytical flowchart for DNA methylation data based on exposure groups.

Figure S3. Analytical flowchart for the association between SNPs, DNA methylation levels, and exposure groups.

Figure S4. Association between SNPs and DNA methylation levels analyzed through quantitative and qualitative models, with different DNA methylation grouping.

Figure S5. Association between SNPs and exposure analyzed by Pearson correlation.

Figure S6. The methylation β value of the CpG probes corresponding to rs11085020 (NFIC), rs199442 (NSF), and rs10947050 (RNF39) under different allele types.

Table S1. Exposure-related metabolites identified in previous studies.

Urine metabolites

Table S2. Pathway analysis results from 45 genes corresponding to CpG probes with DNA methylation levels significantly associated with exposure status in 159 study subjects using the Database for Annotation, Visualization, and Integrated Discovery (DAVID) platform.

Table S3. Pathway analysis results from putting all 865,918 CpG probes with individual urine As, Hg, and V concentrations, respectively, through Gene-Set Enrichment Analysis (GSEA) in 159 study subjects.

Figure S1. GIS map of (A) Yunlin County in central Taiwan and (B) location of high and low exposure areas and petrochemical plants.


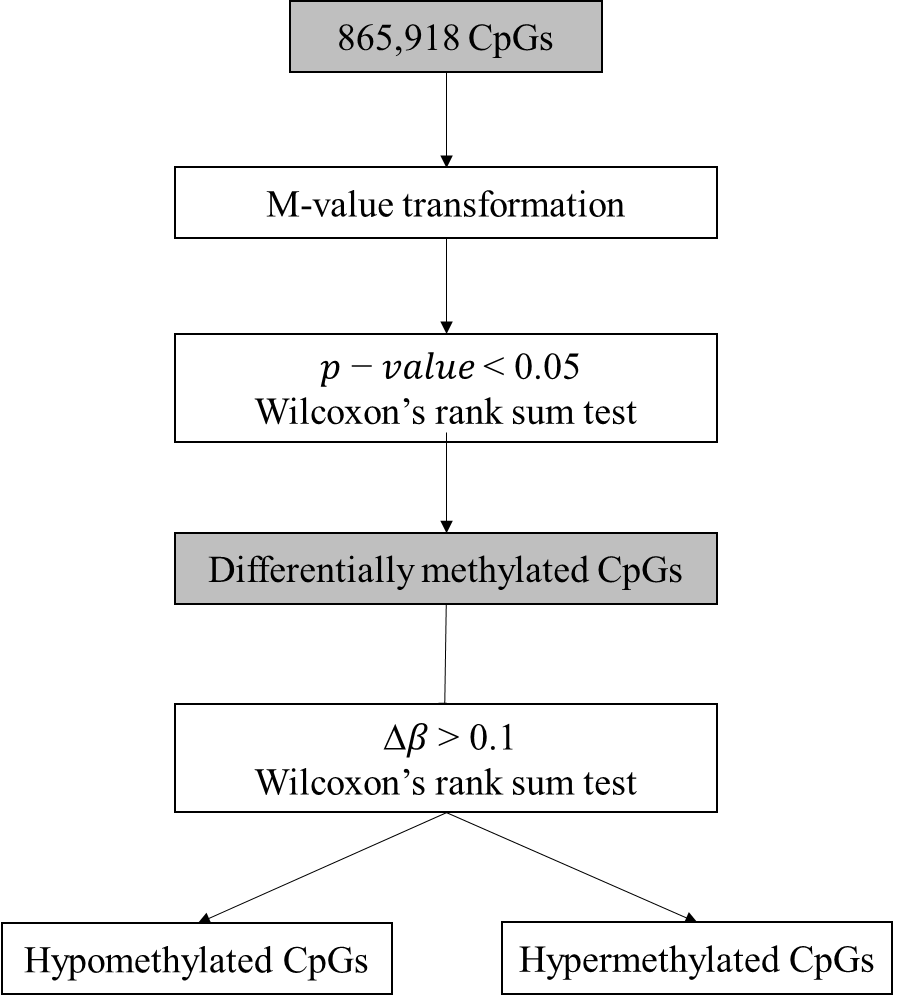


Figure S2. Analytical flowchart for DNA methylation data based on exposure groups.


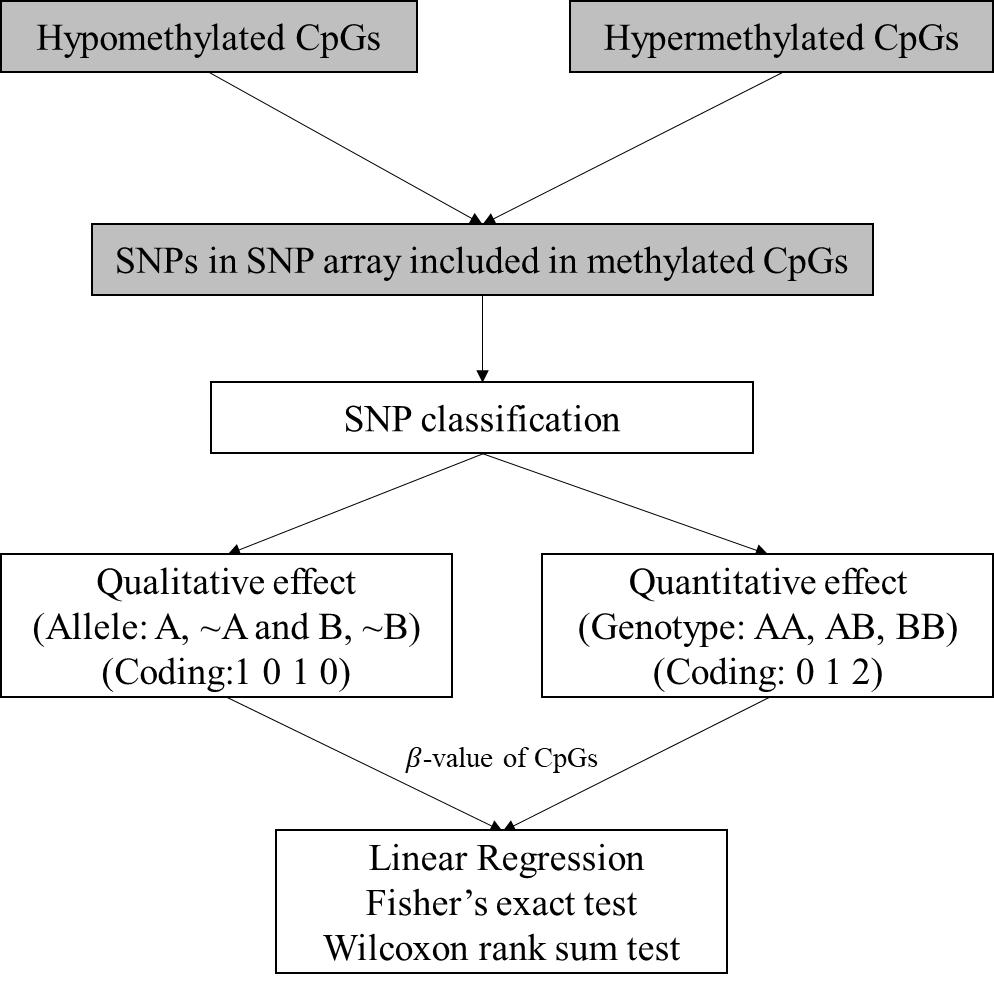


Figure S3. Analytical flowchart for the association between SNPs, DNA methylation levels, and exposure groups.


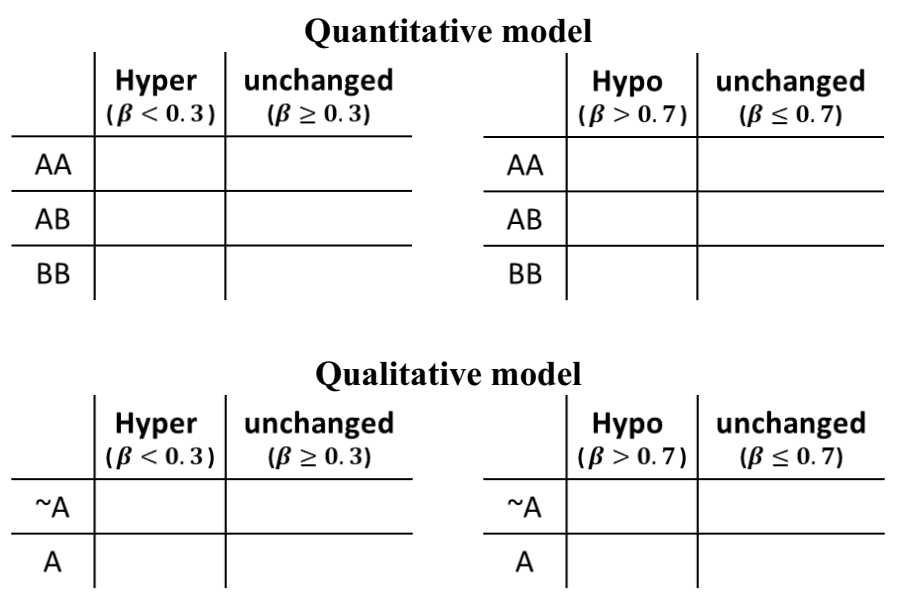


Figure S4. Association between SNPs and DNA methylation levels analyzed through quantitative and qualitative models, with different DNA methylation grouping.


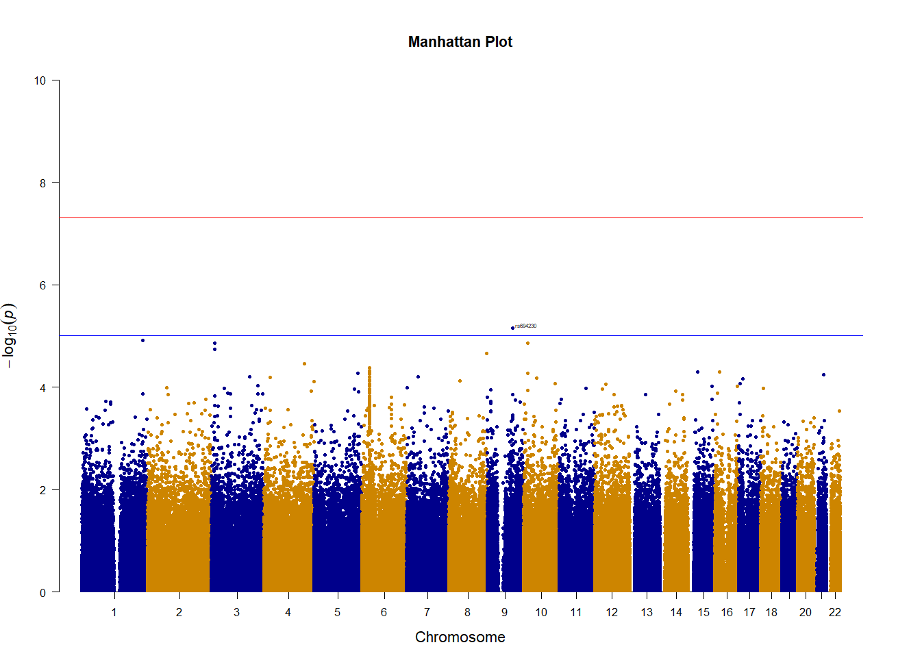


Figure S5. Association between SNPs and exposure analyzed by Pearson correlation.


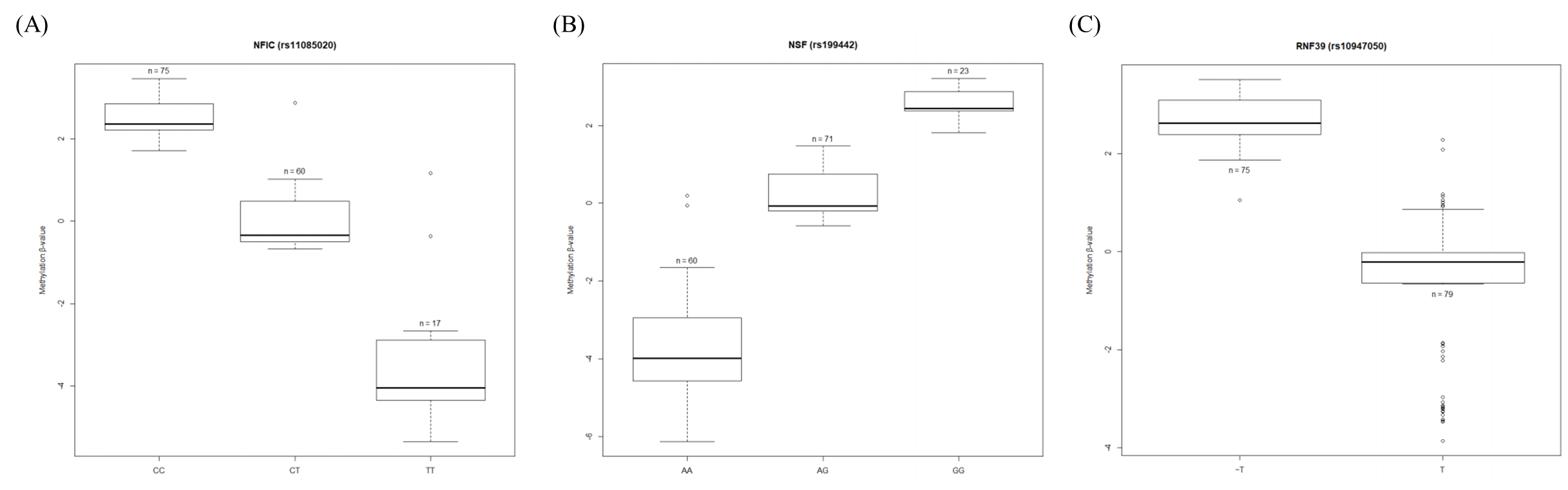


Figure S6. The methylation β value of the CpG probes corresponding to rs11085020 (NFIC), rs199442 (NSF), and rs10947050 (RNF39) under different allele types.

Table S1. Exposure-related metabolites identified in previous studies.

| Urine metabolites  *(Chen et al., 2017)* | Serum metabolites  *(Chen et al., 2019)* | Serum lipids  *(Chen et al., 2021)* |
| --- | --- | --- |
| (S)-3-Hydroxyisobutyric acid | Aspartic acid | LPC (17:0) |
| 1,3-butanediol | Carnitine | LPC (18:1) |
| 1H-Indole-3-acetamide | Inosinic acid | LPC (19:0) |
| 2,4-Dihydroxybutanoic acid | Isovalerylcarnitine | LPC (22:6) |
| 2-Deoxy-L-ribono-1,4-lactone | Ketoleucine | PC (16:0/20:1) |
| 2-Ethylhydracrylic acid | Malic Acid | PC (18:0/18:0) |
| 2-Hydroxyglutaric acid | Octenoyl-L-carnitine | PC (18:2/14:0) |
| 2-phenylpropanal | Oxoglutaric acid | PC (18:2/17:1) |
| 3-Hydroxyhippuric acid | Pyroglutamic acid | PC (18:2/20:2) |
| 3-Hydroxyoctanoic acid |  | PC (18:2/20:5) |
| 4-Deoxyerythronic acid |  | PC (18:3/18:2) |
| 4-Hydroxybenzoic acid |  | PC (20:1/18:0) |
| 4-Pyridoxic Acid |  | PC (20:4/16:0) |
| 5-Hydroxyindoleacetic acid |  | PI 32:1 |
| Acetoin |  | PI 34:3 |
| Alanine |  | SM (d18:1/21:0) |
| Aminomalonic acid |  | SM (d18:1/22:0) |
| Arabinose |  | SM (d18:1/25:0) |
| Azelaic acid |  |  |
| Borneol |  |  |
| Catechol |  |  |
| Cyanic acid |  |  |
| Cyanuric acid |  |  |
| Cyclohexanone |  |  |
| Cytosine |  |  |
| Decane |  |  |
| Diacetone alcohol |  |  |
| Diethanolamine |  |  |
| Dimethyl sulfone |  |  |
| Dimethylamine |  |  |
| DL-Tryptophan |  |  |
| Dodecane |  |  |
| D-Threitol |  |  |
| D-Xylono-1,5-lactone |  |  |
| Fumaric acid |  |  |
| Gluconic acid |  |  |
| Glutaric acid |  |  |
| Glyceric acid |  |  |
| Glycerol 3-phosphate |  |  |
| Hex-2-ulosonic acid |  |  |
| Hippuric acid |  |  |
| Hydroxyphenyllactic acid |  |  |
| Hydroxypyruvic acid |  |  |
| Hypoxanthin |  |  |
| Inositol |  |  |
| L-Alpha-aminobutyric acid |  |  |
| L-Aspartic acid |  |  |
| Leucine |  |  |
| L-Gulonolactone |  |  |
| L-Histidine |  |  |
| L-Phenylalanine |  |  |
| L-Sorbose |  |  |
| L-Threonine |  |  |
| L-Valine |  |  |
| m-Cresol |  |  |
| Mono(2-ethylhexyl)phthalate [MEHP] | |  |
| Myristic acid |  |  |
| N-acetylglutamic acid |  |  |
| o-Cymene |  |  |
| Palmitic acid |  |  |
| Phenol |  |  |
| Phosphoric acid |  |  |
| Picolinic acid |  |  |
| Quinolinic acid |  |  |
| Rhamnose |  |  |
| Serine |  |  |
| Stearic acid |  |  |
| Succinic acid |  |  |
| Sumiki's acid |  |  |
| Thiodiacetic acid |  |  |
| Threonic acid |  |  |
| Tiglic acid |  |  |
| Tiglylglycine |  |  |
| Tridecane |  |  |
| Uracil |  |  |
| γ-Aminobutyric acid |  |  |

Table S2. Pathway analysis results from 45 genes corresponding to CpG probes with DNA methylation levels significantly associated with exposure status in 159 study subjects using the Database for Annotation, Visualization, and Integrated Discovery (DAVID) platform.

| **Category** | **Term** | **Count** | **%** | ***p* value** | **Genes** |
| --- | --- | --- | --- | --- | --- |
| UP_KEYWORDS | Cytoplasm | 17 | 38.6 | 0.006 | NSF, YES1, MYO10, COPB1, HSPB7, HUS1, TSPY4, IL16, BICD2, PDE11A, KIAA0922, MCF2L, RNF39, AHRR, FAM193B, MAD1L1, DFNA5 |
| UP_KEYWORDS | Alternative splicing | 28 | 63.6 | 0.006 | FAM47E-STBD1, HSPB7, AOAH, ANTXR1, BICD2, PDE11A, MCF2L, RNF39, FAM47E, TBC1D22A, NSF, CDK18, PTPRN2, MYO10, HUS1, IL16, SLC2A9, KIAA0922, DCHS2, DLG5, NFIC, RBM41, STT3A, AHRR, FAM193B, RADIL, MAD1L1, DFNA5 |
| UP_SEQ_FEATURE | splice variant | 22 | 50.0 | 0.009 | CDK18, PTPRN2, HSPB7, HLA-B, IL16, ANTXR1, BICD2, SLC2A9, PDE11A, KIAA0922, DCHS2, NFIC, DLG5, MCF2L, RNF39, RBM41, AHRR, FAM193B, RADIL, FAM47E, TBC1D22A, DFNA5 |
| GOTERM_BP_DIRECT | GO:0006890~retrograde vesicle-mediated transport, Golgi to ER | 3 | 6.8 | 0.010 | NSF, COPB1, BICD2 |
| GOTERM_CC_DIRECT | GO:0005886~plasma membrane | 14 | 31.8 | 0.022 | NSF, YES1, MYO10, COPB1, HLA-B, IL16, ANTXR1, BICD2, SLC2A9, KIAA0922, DCHS2, DLG5, MCF2L, DFNA5 |
| GOTERM_MF_DIRECT | GO:0017137~Rab GTPase binding | 3 | 6.8 | 0.024 | NSF, TBC1D22A, BICD2 |
| UP_SEQ_FEATURE | domain:PDZ 4 | 2 | 4.5 | 0.028 | DLG5, IL16 |
| UP_KEYWORDS | Phosphoprotein | 22 | 50.0 | 0.029 | NSF, CDK18, YES1, PTPRN2, MYO10, SERPINA10, MYBPH, IL16, ANTXR1, BICD2, SLC2A9, PDE11A, KIAA0922, NFIC, DLG5, MCF2L, TRAPPC12, RBM41, FAM193B, RADIL, TBC1D22A, MAD1L1 |
| GOTERM_CC_DIRECT | GO:0005737~cytoplasm | 16 | 36. 4 | 0.029 | NSF, YES1, MYO10, COPB1, HSPB7, TSPY4, IL16, BICD2, KIAA0922, DLG5, MCF2L, RNF39, AHRR, FAM193B, MAD1L1, DFNA5 |
| GOTERM_CC_DIRECT | GO:0031527~filopodium membrane | 2 | 4.5 | 0.030 | MYO10, ANTXR1 |
| INTERPRO | IPR001478:PDZ domain | 3 | 6.8 | 0.031 | DLG5, RADIL, IL16 |
| GOTERM_BP_DIRECT | GO:0016192~vesicle-mediated transport | 3 | 6.8 | 0.032 | NSF, COPB1, TRAPPC12 |
| UP_SEQ_FEATURE | domain:SH3 | 3 | 6.8 | 0.037 | YES1, DLG5, MCF2L |
| SMART | SM00228:PDZ | 3 | 6.8 | 0.038 | DLG5, RADIL, IL16 |
| KEGG_PATHWAY | hsa04940:Type I diabetes mellitus | 2 | 4.5 | 0.048 | PTPRN2, HLA-B |
| GOTERM_BP_DIRECT | GO:0006891~intra-Golgi vesicle-mediated transport | 2 | 4.5 | 0.049 | NSF, COPB1 |

Only *p* < 0.05 results are shown.

Table S3. Pathway analysis results from putting all 865,918 CpG probes with individual urine As, Hg, and V concentrations, respectively, through Gene-Set Enrichment Analysis (GSEA) in 159 study subjects.

|  | **Pathway name** |
| --- | --- |
| As | KEGG_ASCORBATE_AND_ALDARATE_METABOLISM |
|  | KEGG_OLFACTORY_TRANSDUCTION |
|  | KEGG_PENTOSE_AND_GLUCURONATE_INTERCONVERSIONS |
|  | KEGG_STARCH_AND_SUCROSE_METABOLISM |
| Hg | KEGG_PROXIMAL_TUBULE_BICARBONATE_RECLAMATION |
|  | KEGG_PATHOGENIC_ESCHERICHIA_COLI_INFECTION |
|  | KEGG_ALDOSTERONE_REGULATED_SODIUM_REABSORPTION |
|  | KEGG_LINOLEIC_ACID_METABOLISM |
| V | None |

Only *p* < 0.05 results are shown.
